# Living and dying between cultural traditions in African & Caribbean Heritage families: a constructivist grounded theory

**DOI:** 10.1186/s12904-024-01503-6

**Published:** 2024-07-18

**Authors:** Joanna De Souza, Karen Gillett, Yakubu Salifu, Catherine Walshe

**Affiliations:** 1 International Observatory on End-of-Life Care, Division of Health Research, Lancaster University, Lancaster, UK; 2 King’s College London, Nursing, Midwifery & Palliative Care, London, UK

**Keywords:** Advance care planning*, Communication, Minority groups*, Ethnicity, Black or African/ethnology, Palliative care, Terminal care*, Adult-children/ethnology

## Abstract

**Background:**

Ethnic differences influence end-of-life health behaviours and use of palliative care services. Use of formal Advance care planning is not common in minority ethnic heritage communities. Older adults expect and trust their children to be their decision makers at the end of life. The study aim was to construct a theory of the dynamics that underpin end-of-life conversations within families of African and Caribbean heritage. This is a voice not well represented in the current debate on improving end-of-life outcomes.

**Methods:**

Using Charmaz’s constructivist grounded theory approach, a purposive sample of elders, adult-children, and grandchildren of African and Caribbean Heritage were recruited. In-person and online focus groups were conducted and analysed using an inductive, reflexive comparative analysis process. Initial and axial coding facilitated the creation of categories, these categories were abstracted to constructs and used in theory construction.

**Results:**

Elders (*n* = 4), adult-children (*n* = 14), and adult grandchildren (*n* = 3) took part in 5 focus groups. A grounded theory of living and dying between cultural traditions in African and Caribbean heritage families was created. The constructs are (a) Preparing for death but not for dying (b) Complexity in traditions crosses oceans (c) Living and dying between cultures and traditions (d) There is culture, gender and there is personality (e) Watching the death of another prompts conversations. (f) An experience of Hysteresis.

**Discussion:**

African and Caribbean cultures celebrate preparation for after-death processes resulting in early exposure to and opportunities for discussion of these processes. Migration results in reforming of people’s habitus/ world views shaped by a mixing of cultures. Being in different geographical places impacts generational learning-by-watching of the dying process and related decision making.

**Conclusions:**

Recognising the impact of migration on the roles of different family members and the exposure of those family members to previous dying experiences is important. This can provide a more empathetic and insightful approach to partnership working between health care professionals and patients and families of minority ethic heritage facing serious illness. A public health approach focusing on enabling adult-children to have better end of life conversations with their parents can inform the development of culturally competent palliative care.

## Background

Ethnic and cultural differences influence patterns of health behaviours around end of life and in particular the take up of current palliative care services [1, 2]. There are a range of factors at play including cultural approaches to advance care planning, end-of-life values, care preferences at the end of life and inequalities in the provision and accessibility of services [3–10].

Despite an increasing take up of palliative care from people of ethnic minorities, advance care planning remains unpopular [11, 12]. Concerns remain about how to honour individual end-of-life preferences and reduce distress and confusion among surrogate decision makers [12, 13]. Fearing causing distress, coupled with a religious view that ‘it is in God’s hands’, and a desire for a collective family based system of decision making, result in fewer conversations about end of life being reported in families of Black and minority ethnic heritage migrant communities [12, 14–16].

In a range of minority ethnic heritage migrant communities, adult-children often play a key role as end-of-life decision makers for their parents [14, 17, 18]. However, few of these children have conversations with their parents about their end-of-life preferences, and there is often poor congruency between elders and children about preferences of care at end of life [13, 19]. In addition, when surveying the relatives of family members of people who have died, having a documented advance care plan resulted in a higher level of unmet mental health needs for people who identify as black [20]. Though the cause remains unknown, Luth & Prigerson recommend caution and further research since advance care planning might differ in effectiveness for different communities. The benefits of such plans may be based on an assumption that control of future care is a good thing and that choice will be available to people equally [21]. Zivkovic’s discussion of temporal dissonance and the risk of advance care planning not only forecasting futures but also potentially foreclosing possible futures indicates the complexity of this practice when used by people of different cultures [21, 22]. A meta ethnographic approach was used to explore existing research on perspectives of older people from Black and minority ethnic heritage about having end-of-life family conversations [16]. Four storylines were constructed:

*My family will carry out everything for me*,* it is trust; No mum*,* don’t talk like that; I leave it in God’s hands; Who’s going to look after us?* Older people acknowledged that while they do not find it comfortable talking about dying, they trusted their children to make the right decisions for them when needed. Some recognised these decisions may be different to the ones they might make for themselves but felt this was not important. However, several found that it was their children who closed down conversations. Children acknowledged they were frightened and found it distressing to contemplate the death of their parent. There continues to be a high level of religiosity in some ethnic minority heritage communities and those religions favoured a perspective of God having a direct influence on the timing of death of people. Lastly there was a growing concern, particularly in the South Asian communities, that family patterns were changing and that children would not be able to play the caring roles that had existed in the past. Recognising a need to change but also keen to maintain cultural traditions, these older people felt a sense of change and disquiet about their own futures [16].

A complex interplay was found between avoidance as protection [14], a world view that God will plan the time, place and nature of death and that children will make the right decisions for their parents when needed resulting in a silence instead of discussions of end-of-life preferences. Our current narrow approaches to the influences of culture in advance care planning could be widened by exploring the perspectives of a broader range of society [6].

More work is needed to explore how standard palliative care interventions work for different communities and the reasons why people’s experiences may vary between different communities [12, 23]. To develop understanding of what helps and what hinders the reduction of distress at the end of life, the aim of this study was to explore what people from African and Caribbean heritage feel about end-of-life care planning conversations within their families and the role these conversations should play.

### Definitions used in this study

Palliative and end of life care are defined differently in different contexts, however they both have at their heart, the alleviation of health related suffering in the face of serious illness [24]. For the sake of brevity, the term palliative care is used to encompass palliative and end of life care. To denote populations who have in their history migrated to become of a minority ethnicity heritage in countries such as the UK, the term minority ethnic will be used. It is acknowledged that this is complex and risks assuming a conformity that does not exist [25]. Care is needed to recognise these limitations.

The view of what constitutes a good death remains a ‘contested space’ [26]. Much of the expectation of good dying in the literature is informed by evidence gathered from predominantly white communities, rather than a more plural global perspective [26–28]. When facing a terminal diagnosis and seeking health care support, independence, individualism, autonomy, fear of relentless efforts extending poor quality life, choice and veracity are the values that shape the provision of palliative care [27]. These values take the shape of open discussions about prognosis and options, autonomous decision making in the form of formal advance care planning and acceptance of input from health care professionals focused on symptom management and comfort rather than cure. Rates of completion of formal advance care planning remain lower in all minority ethnic groups [29]. As with all cultural processes, advance care planning is a field that changes constantly due to prevailing influences. However, whilst completion of care plans amongst black minority populations is rising, they remain low and indicate a higher preference for continuing ‘aggressive’ care than white populations [15, 30]. Among most minority ethnic populations, choosing a collective family decision making model remains an expressed preference. This is a complex matter and it is important to explore preferences expressed in some detail as there can be a translation gap due to different health literacies, they may not reflect the values of the person expressing them, but more disguise concerns they have regarding treatment choices [8].

## Methods

### Research aim

To construct a theory of the dynamics that underpin end-of-life conversations within families of African and Caribbean heritage.

### Study objectives:

1. To explore the perceptions of older adults and their adult-children about having family conversations around end-of-life preferences.
2. Using constructivist grounded theory methodology to develop a theoretical understanding of these experiences.
3. To develop ways of enabling health care practitioners to connect with this theory when working with families.
4. To consider strategies to engage communities to consider the significance of this theory and how it can be used to influence the development of culturally sensitive approaches to having end-of-life family conversations.

### Design

A subjective and interpretivist approach of constructivist grounded theory using the model developed by Charmaz [31] was chosen to construct a theoretical understanding of people’s experiences and perceptions in the form of a descriptive theory. Grounded theory relies on the gathering of rich thick data [32]. Focus groups are the data collection approach for this study because capturing a group discussion provides co-created knowledge by the group than a ‘singular truth’ about the research question [33].

### Population

The study population was people of African or Caribbean heritage living in the UK. The inclusion criteria for the study were:

Adults aged over 60 years who identify as of African or Caribbean heritage, live in the UK, and have children or next generation family members over 18 years old.

OR

Adults over 18 years of age who have a parent or other family member over 60 years old, who identify as of African or Caribbean heritage and live or have lived in the UK.

### Sampling

A purposive approach to sampling was deployed, with potential participants given the opportunity to respond to a call for participants via various means. The research team discussed theoretical sampling following the analysis of data from the third and fourth focus groups. Emerging data indicated there might be gendered influences on perceptions and experiences and some of the older adult child participant’s parents who had already died. The decision was taken to capture the voice of younger people who potentially might be having earlier conversations with their parents and to include male voices in focus group 5.

### Recruitment

Study recruitment was mainly targeted at communities in South London where there is a high proportion of people from an African or Caribbean heritage. We did not actively recruit people with an established illness, however nor were they participants excluded on that basis. The study was advertised in community settings with posters displayed in churches, a university, a primary school, a shop, and pub in areas with high populations of people from African and Caribbean heritage and a Caribbean Hindu temple. Social media (twitter, Instagram, Facebook) were also used to inform people of the study, together with a university research volunteer recruitment circular and an internal organisational Yammer group.

### Data collection

The first two focus groups were face-to-face and the last three were online due to the need for social distancing during the COVID- 19 global pandemic [34]. The primary researcher moderated all the focus groups, but a second researcher sat in on the groups as an observer who made notes of what was said and the interactions taking place. Each started with an introductory time of informal socialising to allow participants to meet prior to the focus group. This was more successful in the face-to-face groups than those run online [34].

Participants in the groups discussed the following questions:

1. What do older people from African or Caribbean heritage want to plan for at the end of life?
2. What role do they think their children should play in the decisions about their health care at this stage?
3. How do older people and adult-children within families find talking to each other about end-of-life care planning?

### Data analysis

The focus group sessions were recorded and transcribed using data analysis software NVIVO version 12 [35]. Each script was coded using line by line initial coding in-vivo coding using the direct words of the participants which retained the voice and language of the participants through the analytic process [31, 36, 37]. Following this initial coding, the codes were reconsidered using process coding where gerunds or action words are employed to capture the dynamics of the experiences being expressed (Table 1).

**Table 1:** Types of initial coding

Initial line by line coding using In-vivo coding	he’s always said he’s prayed and asked that God takes him before
Sensitizing concepts	*Umm no we’ve not talked about end of life or anything like that*
FG1 process coding	Process code - Trusting *God*

Conversation dynamics were also noted and analysed using sociograms [34, 38]. Care was taken to capture what is stressed by participants in the discussion. Wider discussion with the other research team members led to axial codes and category development (Table 2) [31, 37].

Once this was completed for the first two focus groups, initial coding was followed by focused coping where significant or frequent initial codes were considered by the primary researcher (JDS) and researcher observer (KG) along with both the research field notes to see what made most analytic sense for categorising data (Table 2). This process was documented using a Microsoft excel document. This was shared and verbally discussed with the wider research team (CW & YS) along with the initial group transcripts.

**Table 2:** The process of coding from initial codes to constructs

Initial codes	Process/ Focused codes	Constructs
friends’ parents are dying so we talk about things I talk with mum because her mum needs a lot of care now	Learning by watching	Watching the dying of another prompts conversations

The constructs were derived from the categories that reflect the grounded data and a process of reflecting using the processing of memoing to reflect on the ideas as a research team, these are discussed in the results.

### Ethical considerations

Careful consideration was given to psychological safety of participants being asked to share personal experiences and perspectives around emotive issues such as the future death of loved ones. All participants received information about the study and at least 48 h to consider whether they wanted to take part. Verbal and written informed consent was obtained. All responses were anonymised, and participant’s heritage ethnicity was referred to as African or Caribbean when attached to in vivo quotations to minimise individual attribution. Research ethics committee approval to conduct the study was granted by the research ethics committee at Lancaster University FHMREC18 (August 2019). The study was considered low risk by King’s College London and allowed to proceed. In July 2020 a modification request was approved to move data collection online.

## Results

Twenty one participants were recruited into 5 focus groups between 2020 and 2023 (Tables 3 and 4). Each focus group lasted between 60 and 90 min.

**Table 3:** Sample characteristics

Characteristic	Participants (*n* = 21)
Gender	M = 2 F = 19
Heritage	Ghana *n* = 3 Nigeria *n* = 4 Kenya *n* = 1 Jamaica = 7 Trinidad = 1 Grenada = 1 Mixed Nigeria/Ghana) = 3 Mixed English/Caribbean = 1
Age – Adult Grandchildren Adult-children Elders	20–29 *n* = 3 30–40 = 0 40–59 = 14 60–70 = 3 > 70 = 1
Recorded Religion	Christian = 14 Muslim = 1 None recorded = 6

**Table 4:** Focus group participants

Group	Range	Heritage	Context
1	Adult-children between 50-60y 5 women	Ghana, Nigeria, Kenya, Jamaican	Professional women working in one higher education institution but in different roles
2	Older adults, between 65-80y 3 women, 1 man	Ghana, Nigeria, Jamaican	All attending same church
3	Adult-children between 45-60y 6 women	Ghana, Nigeria, Jamaica Grenada	Professional women from a variety of backgrounds
4	Adult-children between 45-55y 4 women	Ghana, Nigeria, Trinidad	3 Professional women, one nonprofessional woman who lived independently but was visually impaired,
5	Adult grandchildren between 28-30y 2 women, 1 man	Ghana, Nigeria, and mixed-race Scotland/ Nigeria	Young professionals

### Developing a theory

Data collection and interpretive synthesis of the data collected led to the development of constructs of a substantive grounded theory of Living and Dying between Cultural Traditions in African & Caribbean Heritage Families. In this theory, the constructs, and related categories of how a family’s habitus regarding end-of-life conversations are shaped are presented (Table 5). Being part of multigeneration families who are assimilating into a culture different to the one in which the older people may have been born experiences are a core element reflected.

**Table 5:** Constructs and related categories

	Constructs	Categories
1	Preparing for death but not for dying	We are planners of funerals but not of care
2	Complexity in traditions crosses oceans	Talking about cancer and dying is taboo
3	Living and dying between cultures and traditions	I am a London girl; things are different in Ghana
4	There is culture, gender and there is personality	Intersectional influences on negotiating family decision making, who speaks to whom about what and when
5	Watching the death of another prompts conversations	I talk with mum because her mum needs care now
6	Hysteresis	Inertia and change

**Fig. 1:**
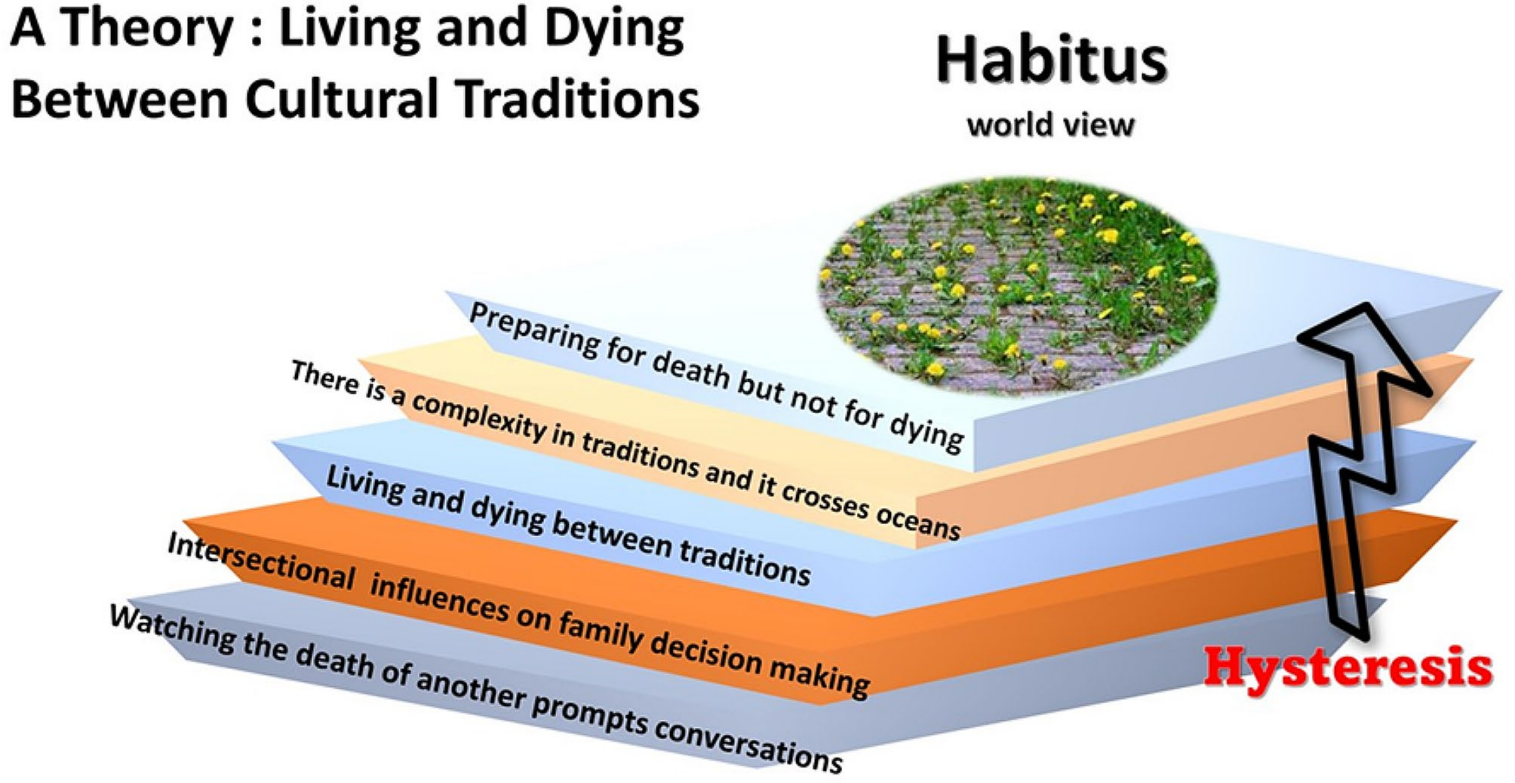
A conceptual model of the theory of living and dying between cultural traditions

Analysis of the conversations between the participants suggest that a complex set of life experiences shaped their visible habitus. All the participants had lived in the UK for a substantial period, some were born in the UK. However, their views and experiences of conversations about dying within their families was very connected to both their current life situations and to the practices, values, and beliefs of their heritage countries. They all also experienced a sense of hysteresis [39] or pull between familial cultural norms and practices and the cultural norms of the normative populations in the country in which they now lived (Fig. 1).

### The constructs of the theory

#### Preparing for death but not for dying; We are planners of funerals but not of care

Across cultures many people in older generations prepared for their funerals. In this study several participants spoke of their parents having financial funeral plans, although the older adults did not disclose this. There was an openness around funerals and funeral planning. However, as the groups discussed these financial arrangements there was a developing awareness that much of the conversation centres around after death planning. There was agreement that the process of dying was rarely discussed:

*It feels different in Ghana it seems so public there*,* there are posters about funerals coming up*,* people talk about it*,* funerals and memorials are more about celebrating the life lived than mourning. (Adult child*,* African)*

*In Grenada*,* …. they have big billboards up …. it has a photo of the person and all the family (Adult child*,* Caribbean)*.

*And we’ve certainly talked about death and funeral plans and all that sort of thing. But I feel talking about death and dying is still very much taboo and not really spoken about. It is something I would like to visit with mum. (Adult child*,* African)*

The older adults in the study had great concern about having everything sorted out to reduce the stresses on their children. They spoke about how their children silence conversations:

*When I do talk to my daughter*,* I start to say*,* when I am dying …she says*,* no mum don’t talk like that…. I feel they are all frightened of losing me…so I don’t talk about it because I don’t want to upset them*,* but I make my own plans… (Older Adult child*,* African)*.

These are cultures who have strong open traditions around marking death, visible and shared with the whole community. People are now having to compromise these to fit in with new cultures where funerals are less visible and generally private. The conversational opportunities these intergenerational community funeral events presented are reduced and a fear of talking about the death of a parent results in a hiatus of conversation between one generation and the next.

#### Complexity in traditions crosses oceans: talking about cancer and dying is taboo in our culture

Part of this lack of discussion about dying and illness may be associated with fear and taboo around serious illnesses and particularly cancer. Many of the participants who shared their family stories, felt this resulted in a lack of open family disclosure:

*When I was diagnosed*,* I remember having a discussion with my mother about it. And*,* you know*,* the kind of distress it caused meant that I was never*,* ever able to have a conversation with her again*,* it was because it was cancer and she was petrified*,* It was a real shame because*,* you know… your mom’s your confidant (adult child*,* Caribbean)*.

*My brother has cancer*,* he and I talk… but we don’t talk about it with mum and dad. Dad had the same cancer and now …. so there’s a bit of self-preservation with that. (adult child*,* African)*

This sense of fear and taboo remains strong even when families have been living in the UK for a while. As one participant raised this, others recognised that this was a shared experience:

*So it’s interesting that you’re saying that because a close relative of mine who was very ill but didn’t say anything and kept it to themselves*,* I don’t know if it’s a Caribbean thing…. when you kind of think to yourself*,* why did I not know*,* why is it that I’m here in the UK you’re over there in Jamaica. Why am I hearing now*,* right now. Because it’s like if I had known I would have been able to go over (adult child*,* Caribbean)*.

This concept has overlaps with the concept of living between traditions (concept 3) and the hysteresis or tension that exists when one cultural practice is very different to another (concept 6). We consider the impact of this on family conversations as we explore the next layers of this constructed theory.

Several participants had experienced serious illness or the death of a younger person within the family. In their discussions they discussed the prevailing funeral tradition of parents not attending the funerals of their children. Although participants discussed how difficult this might have been for the parents concerned there was also an acceptance that this was an important aspect of culture that needed to be respected:

*That’s right*,* yeah…you know my nephew who died recently*,* he died over here and it was sudden so although we took him home*,* his parents never saw him*,* they did not came to the funeral (Adult child*,* African)*.

*Yes*,* I had an older sister who passed but they never saw her and they weren’t at the funeral because it is not done (Adult child*,* Caribbean)*.

There were discussions about how experiences caused a change in what people wanted, that this was perhaps a tension for people, and so not openly discussed between family members. Participants, both the older adults and the adult-children spoke about situations where they felt they were *Living and dying between cultures and traditions*: The category of *I am a London girl*,* things are different in Ghana* indicated this changing perception of who they were and the challenge of what traditions to keep up with and what different ways of doing things may be more in tune with how they currently perceive themselves. Some of the adult-children could see that in their parents as well:

*I’m very much westernized*,* a London girl. And in Ghana*,* things happen differently*,* there would be many traditions like painting the house and I don’t think mum would want that*,* she’d prefer it painted now while she can enjoy it …But the one thing … is…. previously she always said that when she died she… she’s talked about….being buried in Ghana. …. there’s a family plot or whatever…. But when my sister died*,* she did make a comment that…if I die either I don’t mind being buried here or in Ghana. . (Adult child*,* African)*

*My mother bought a house*,* she had always planned to go back to retire . you know. but my dad he didn’t want to leave the grandchildren and everything…(Adult child*,* African)*.

*And yeah*,* it’s like that that’s in my family. That has been the dilemma. One parent wanting to go back and one parent just not wanting to go back ever …(Adult child*,* Caribbean)*.

Finding themselves part of families with ties to more than one place, each with different sets of rituals and values made the universal experiences of generational change more complex. This is additionally complicated around death rituals which are tied to the marking of respect which is an important value in many cultures.

#### There is culture, gender and there is personality: who discusses what with whom

Participants acknowledged that conversations about future care preferences were rare in their families both between and within generations. There was an expectation that adult-children would make future care decisions as best they could, without having discussed preferences.

*I remember from a young age*,* my mom doesn’t say so much now…. You’re the youngest and it’s your job to look after us when we’re old … (Adult child*,* African)*.

*So if she becomes less well… what do you think she will want … well that’s going to be my decision. She’s…. She’s not going to discuss it. … it’s just whatever I decide. That’s how it is. (Adult child*,* Caribbean)*

Parents tried to protect their children from having to feel responsible for them:

*We don’t want to bother them. If we can do something for ourselves*,* we will do it. sometimes we need help with this and that*,* but we don’t want to bother them (Older Adult child*,* African)*.

When family members are unwell, care decisions and treatment decision making can be particularly complex when family members are living in different countries, particularly when the older person was unwell and had limited cognition. Some participants spoke about this:

*Yeah. And I think it’s a culture thing*,* because I remember when I lost my brother and he was in the States. We had to take his body back home. And we were having a conversation about where he was going to get buried and you know… my uncles were at the forefront… we it’s like we have no say. (Adult child*,* African)*

However, as the discussions developed it becomes clear it is much more complex than that, more about proximity and funding.

*… when my dad died*,* he was living in the US with my half-sister*,* she made she made all the decisions …kind of you know*,* he was over there. We were over here. oh*,* my goodness me. it was it was very*,* very*,* very*,* very*,* very*,* very tricky (Adult child*,* Caribbean)*.

*And I think there is a there is a power dynamic going on because frankly*,* my dad or the siblings who live in the UK they send back money quite often. So . usually they don’t look after grandma…. But in terms of making decisions*,* I think they have a lot of say because it seems like if she… needs to go to the hospital*,* he(participant’s father) gets informed …. (Adult grandchild*,* African)*

Negotiating and planning and funding when family live in different countries adds layers of complexity to an already emotional and difficult process. Participants spoke of the value of modern communication aids like WhatsApp in helping to negotiate in these challenging times. Participants indicated that WhatsApp allowed all parties to be part of the conversation, including the participant who was visually impaired:

*Oh*,* my God*,* it’s amazing. A revolution. It really has. You can make decisions on WhatsApp. You know*,* …. We were deciding when the headstone for my brother actually this week and the decisions*,* the discussions were so smooth on WhatsApp. So*,* it really has helped (Adult child*,* Caribbean)*.

Lastly for several participants being part of these focus groups highlighted for them conversations they had not had but wished they had or would like to have in the future:

*Yeah. My mom passed quite a while now*,* 18 years ago*,* and it was very sudden. So*,* there was no conversation with regards to what was going to happen. And we didn’t know what conversation she had with the family there…*,* that she wanted to be buried in Ghana. So*,* it was a compromise*,* she was buried in Nigeria so she could be beside my dad*,* I still kind of feel bad about it because that was a wish and it wasn’t actually done (Adult child*,* African)*.

*It concerns me now that we’re talking about it*,* that I hadn’t had that conversation with my sister myself (Adult child*,* Caribbean)*.

As part of the theoretical sampling the final focus group was with younger people who had living grandparents. Analysis of their reflections along with the previous conversations with adult-children and older people resulted in this final concept: *Watching the death of another prompts conversations*: *I talk with mum because her mum needs care now*. So much of what individuals do is borne out of the way in which they have been culturally nurtured and develops from watching role models such as parents or other aspirational societal members. Watching a family member, a friend or a friend’s parents go through life events like dying, often stimulated conversations about what went well, what was difficult and perhaps what they would want for themselves or their parents when they die.

*I think you don’t realise just how much care someone elderly can need unless you’re in the scenario of caring for them …So mum and I … we have had that conversation recently*,* but before my grandma needed so much care*,* we’d never had that conversation (Adult grandchild*,* Caribbean)*.

Many of the older people and adult-children in this study had been living in another country when their parents or grandparents had died. The impact of this was that they saw only a glimpse of what went on, resulting in a loss of an opportunity to reflect on how it may be for them when they were dying:

*Yes*,* mum was often in Ghana*,* she went to look after him …. But I didn’t go (Adult child*,* African)*.

People of the generation who are now approaching the end of life themselves and their adult-children have often only had fragmented opportunities to be present at the deathbeds of the generations before them. This rehearsal and potential opportunity to consider what they may want for themselves if in a similar situation and to discuss this with other family members around the death bed has been lost.

##### Hysteresis; Inertia and change

Threaded through all the concepts of this theory, is the concept of hysteresis [40]. Hysteresis describes the lag or a miss-alignment in cultural values as part of the process of cultural adaptation [41, 42]. As families assimilate into new cultures there is a tension between what holds, in terms of existing values, beliefs, and rituals and what changes. This tension is sometimes shaped by the power dynamics of both the heritage communities, the host culture or how cultural and symbolic capital is distributed [43].

Captured in the layers of cultural experiences was a sense of change, either between participants and their parents or children or even within their own experiences over time. When there are limited conversations between people and a lack of cultural curiosity into the perceptions of others [44], particularly amongst health care professionals, this cross cutting concept of hysteresis and differences in the way experiences are experienced and understood can be hidden.

## Discussion

This study uses an inductive focus group approach to explore the perspectives of older adults and adult-children of African and Caribbean heritage on having family conversations about end-of-life preferences. A constructivist grounded theory approach was taken to analyse the empirical data collected and to construct a theory of living and dying between cultural traditions in African and Caribbean heritage communities. There is a focus on the perspectives of adult-children, as at the very end of life adult-children are often the end-of-life decision makers for their parents in these communities, and the use of advance care planning is low. The goal of this theory construction is to promote a deeper understanding for health and social care professionals, of the complex underpinnings of diverse cultural perspectives and how experiences of migration impact planning and discussing of key social processes such as aging and dying. Cultural practices become particularly important at significant times such as approaching the end of life (Fig. 1).

Oscillating values and preferences create tensions [21, 41, 42]. Norms and values of the cultures in which people are currently living sometimes jar with those of their heritage cultures. Families are forced to seek ways of living between these tensions. There is a sense of hysteresis or ‘being pulled between’ as families navigate illness and dying when family members live in different places in the world [8, 9]. Often it is simply who lives where that determines who makes the decisions. Geographical location influences the role family members play in making the healthcare decisions when an older family member is coming towards the end of life, for example being the provider of care or being the provider of the means with which to buy the care. Some traditions and cultural practices are cultural assets [6], such as the of openness experienced in both African and Caribbean cultures around funeral planning and celebration. Even for families where whole generations have been born in their country of residence, migration has an impact on cultural learning about death and dying which results in them choosing a different approach to engage in planning for the end of life.

An openness about families attending community funerals and so the exposure to burial rituals throughout the life course is a positive aspect and a cultural asset [6] of both African and Caribbean culture that was celebrated by the study participants. It is on such occasions people are exposed to cultural norms, values and rituals. These multigenerational events offer opportunities for conversations around family practices and plans. Funeral planning is one of these opportunities. More interventional research is needed to embrace the opportunities to think and plan around people’s own values and end-of-life preferences when attending the funerals of others. Co-produced research with both funeral directors and faith leaders who conduct funerals maybe helpful to consider how both discussing care preferences with family could be part of funeral planning [6, 45, 46].

A new concept constructed from the findings of this study is ‘watching the death of another prompts conversations’. A younger participant in the study spoke of how watching her mother care for her grandmother provided an opportunity to discuss her mother’s own preferences. Both the participant and her mother had not realised how complex end-of-life care decision making can be before this experience. Many participants in the study talked of grandparents who died abroad, making that process ‘invisible ’ [47]. Both the older adults living in the UK and their children had limited opportunities of learning-by-watching either in person or vicariously, how the dying of older generations looks and is managed. This has a major impact on their learning about what decisions need to be made when.

Health care professionals’ understanding of this phenomenon, can help them to have greater insight into why dying maybe a taboo for a generation who have missed observing older generations dying. Offering more information about what the dying process and decisions that will need to be made early in discussions about deteriorating illnesses is helpful for most relatives of the dying, however exploring previous experiences and being sensitive to the impact of migration histories on these displays cultural insight. Promoting discussion around decisions, and being a compassionate companion on this new road, can help to provide a space where individuals and families can think and talk about decisions before they need to be made. These are often complex family discussions [42, 48].

Study participants valued the way that WhatsApp Messenger family groups (mobile phone encrypted instant messaging service) facilitated family communication. Many migrant families, now split and living in different geographical places, use social media forums to navigate and communicate complex decisions around burial practices and end-of-life decision making for the seriously ill. This is an area that needs development in palliative care to harness a wealth of opportunities for all communities. Encouragement to use sharing platforms like WhatsApp to enable open discussions maybe helpful when families reside in different locations.

Most of the adult-children participants in our study had experienced the death of a sibling, had a sibling facing a serious illness such as cancer or had experienced a life threatening illness themselves. These are important learning experiences that may leave difficult legacies. Like previous studies, participants discussed how some family members, particularly elders, continued to hold cultural values and beliefs, some fuelled by high levels of religiosity, that resulted in stigma and fear of being associated with cancer [15, 42, 49, 50]. The adult-child participants felt caught in a hysteresis where felt they could only have more open illness related conversations between themselves, but not with older relatives for whom it was taboo [43]. Such cultural positioning is not easy to change or to navigate for families. This creates an element of collusion that can be stressful for all involved. Adult-children may appear ‘difficult’ when trying to manage the fears of their parents or their own fears of losing a parent to the sometimes naïve or ethnocentric health care professional who is foisting a culturally foreign approach to care. A sensitive approach is required to enable more open conversations in a way that retains respect for cultural values recognising the need to understand family histories and migration experiences [42]. Migration leads to hysteresis, pulling between different cultures, where some aspects of heritage cultures are celebrated and retained, but other aspects lead to tensions and mixed expectations, little of which is discussed openly within families or with health and social care professionals. The fracturing of observed and lived experiences of caring for dying people because of migration has an impact on the planning and preparation for dying of the first-generation immigrants and their children.

Much of the existing literature exploring the perspectives of minority ethnic heritage groups focuses on barriers and suggests that language issues and a lack of health literacy cause a reluctance to engage with a normative idea of palliative care and advance care planning developed in majority white non migratory populations. Our study uncovers a much wider complexity of different life experiences from which people develop a habitus or world-view from which they approach subjects such as end-of-life family conversations and decisions making. Exploring and understanding these can be fundamentally important when working with families going facing loss.

### Implications for practice

There is a growing awareness of the impact of structural racism in palliative care [3, 10, 42]. This includes ongoing racial attitudes in the form of symbolic and social violence [51, 52] such as when the expectations developed by one community can be imposed on another reducing access and equality of experiences of healthcare when dying. Greater recognition is needed of the need to shift to a new order of palliative care and related ideas that recognises the need for intersectional structural change and a diversity of approaches that embrace a wider set of cultural ideas [6, 53, 54].

New relational and more individually nuanced models of palliative care are emerging with a focus on communities as sources of invaluable support [55, 56]. For health care professionals working with people with deteriorating health conditions, the need to recognise the role of adult-children in end-of-life decision making, particularly in many African and Caribbean heritage families, is imperative. The findings of this study provides further evidence to the calls to develop more family centred models of advance care planning [6, 12, 57]. Enabling opportunities for better family discussions around end-of-life preferences may help to break down the fears of both adult-children and their parents of burdening the other. All health care professionals working with people with life threatening conditions need to facilitate a better awareness by a range of family members of end-of-life illness processes and the decisions that may need to make in the future, as part of early care planning.

Finding ways of increasing community engagement with dying that perhaps has been lost as funerals become more private events may be useful to explore. Engaging with wider societal influencers such as authors and film makers to ensure dying is included in mainstream depictions of life and opportunities are made to consider and discuss these before the time arrives for families to be making these decisions in acute situations [58].

Clinicians need to offer culturally competent care. First steps in achieving this is having a cultural curiosity, and cultural humility. Knowledge and understanding of the world views of others makes it easier to communicate and reduces potential fear of things that feel different [59]. Exploration of previous serious illness and death experiences allows the clinician to build on these. Advance care planning may need to include consideration of the complex needs of families living in different countries.

### Implications for future research

Exploring different voices helps to create a wider understanding of how different communities interact and their perceptions about the value and nature of family conversations concerning end-of-life preferences. Despite active attempts, this study failed recruit many male participants, further research is required to understand how men in these communities’ experience end of life care and decision making.

To enhance advance care planning at the end of life, it is crucial to conduct further research that builds upon these new insights and explores the best ways to facilitate open conversations for older adults and their families. This should include developing strategies that address the specific emotional, cultural, and communication barriers they face, ensuring that their preferences and values are respected and integrated into care plans.

### Limitations of this study

This was the first study to explore the voices of people of African and Caribbean heritage living in the UK on this topic of end-of-life family conversations and the generosity of their disclosures and insights offers a rich picture into their experiences. This is a small snapshot of a much larger complex process of living and functioning in society where social capitals influence experiences. The insights gained in this study came from people who felt able to discuss issues such as end-of-life conversations. There was a lack of sociological variation, nearly all the participants were graduates and there only two men in the sample. Designing and conducting a study feels accessible for people from a broader spread of society would offer greater insights and possible new ways of working in this area of end-of-life family conversations and general death anxiety.

In conclusion, this grounded theory of living and dying between cultural traditions promotes an understanding of the impact of the migration identities of people of African and Caribbean heritage that are formed around having end-of-life conversations within families. This understanding is helpful in developing new approaches to the practice of palliative care and communication approaches which work better for a culturally diverse population.

## Data Availability

No datasets were generated or analysed during the current study.
